# Enhancing Mechanical Properties of Corn Bran Arabinoxylan Films for Sustainable Food Packaging

**DOI:** 10.3390/foods13091314

**Published:** 2024-04-25

**Authors:** Abdulrahman Alahmed, Senay Simsek

**Affiliations:** 1 Department of Food Science and Nutrition, College of Food and Agricultural Sciences, King Saud University, Riyadh 11451, Saudi Arabia; abalahmed@ksu.edu.sa; 2 Cereal Science Graduate Program, Peltier Complex, Department of Plant Sciences, North Dakota State University, Fargo, ND 58102, USA; 3 Whistler Center for Carbohydrate Research, Department of Food Science, Purdue University, West Lafayette, IN 47907, USA

**Keywords:** arabinoxylan, biodegradable films, tensile strength, tear, puncture

## Abstract

Arabinoxylan (AX)-based films can improve the mechanical characteristics of biodegradable materials when utilized for food packaging. However, the mechanical properties of AX films for food packaging applications require thorough investigation to establish their viability. In this study, AX was extracted from corn bran coproducts of dry-milling (DCB), wet-milling (WCB), and dried distiller’s grains with solubles (DDGS) using an acid–alkali method. Packaging materials were produced using these AX extracts, each combined with laccase and sorbitol, forming the basis for three different films. These films were then modified by immersing the surface in a lipase–acetate solution. We evaluated their mechanical characteristics, including thickness, tensile properties, tear resistance, and puncture resistance. The thickness and tensile properties of the modified AX films derived from DCB and DDGS showed significant improvements (*p* < 0.05) compared to the unmodified AX films. In contrast, the modified AX films from WCB showed no significant changes (*p* > 0.05) in thickness and tensile properties compared to the unmodified WCB AX films. A significant increase in tear resistance (*p* < 0.05) was observed in all modified AX films after immersion in the lipase–acetate mixture. While puncture resistance was enhanced in the modified AX films, the improvement was not statistically significant (*p* > 0.05) compared to the unmodified films. The presence of hydroxyl (OH) and carbonyl (CO) groups on the surfaces of AX films from DCB and DDGS, modified by the lipase–acetate solution, suggests excellent biodegradability properties. The modification process positively affected the AX films, rendering them more bendable, flexible, and resistant to deformation when stretched, compared to the unmodified AX films.

## 1. Introduction

Corn is a cereal also known as maize, and mature corn is referred to as dent maize. One of the major byproducts of both dry-milling and wet-milling operations is corn bran (CB), which has a low commercial value. The bran obtained from dry-milling and the bran from wet-milling have different appearances, such as color and various chemical compounds. The corn dry-milling process includes tempering, conditioning in warm water, heating, drying, grinding, and then sifting through multiple sieves by a mill, which rubs the corn particles to remove the germ and bran, resulting in endosperm flour [1,2]. In contrast, the corn wet-milling process separates the corn grain into the kernel’s main chemical compositions of starch, protein, oil, and fiber-bran. This operation comprises several steps: steeping in 0.2% sulfur dioxide (SO_2_), drying, milling into coarse grounds, separating the germ, grinding into finer grounds, separating the WCB, removing gluten, washing, and sifting into starch flour [3,4].

Bran is rich in fibers, mainly cellulose and hemicellulose. Chemically, the content of CB ranges between 100 and 130 grams per kilogram (g kg^−1^) of protein, 90 and 230 g kg^−1^ starch, 20 and 30 g kg^−1^ lipids, 20 and 15 g kg^−1^ ash, and 200 g kg^−1^ cellulose [5]. Hemicellulose mainly comprises heteroxylan, which is present in the cell walls and accounts for 500 g kg^−1^ of the CB [5,6]. AX is the main component of the heteroxylan found in CB. Moreover, while AX is found in the corn bran, endosperm, and germ, the ratio of arabinose to xylose (A/X) in the AX present in each part of the corn kernel differs [6,7,8]. In other words, the two major fibers of AX found in the corn kernel, bran fiber and endosperm fiber, are characterized by their unique arabinose/xylose ratios.

DDGS are those grains, including corn, that have a dense nutrient coproduct from dry-milled ethanol production [9]. Unlike dry- and wet-milling, the DDGS process does not only reduce the grain particle size but it also includes the specific ethanol production pathway in the conversion of starch to ethanol. DDGS production from maize grains includes the following steps: grinding from 3 to 5 millimeters (mm), slurrying by adding water, liquefying by amylolytic enzymes, fermenting, distilling, centrifuging, and drying [10,11,12]. Most importantly, the solution is fermented by yeast, *Saccharomyces cerevisiae*, which converts sugars to ethanol at 32 Celsius (°C) for 12 hours (h) [12,13]. Ethanol is then collected by distillation columns, and the remaining fluids and solids are called whole stillage, which is centrifuged to remove coarse solids from the liquid [9]. The coarse solids are called wet cake and are subjected to a drying process to produce DDGS. DDGS are mostly used for animal feed that contains 30 g kg^−1^ protein, 15 g kg^−1^ cellulose, and 20 g kg^−1^ hemicellulose; half of the hemicellulose is AX [14,15].

AX is a non-cellulose polysaccharide mainly localized in the cell walls and the aleurone layer surrounding the endosperm of corn grains [7,16]. AX is mainly made up of two monosaccharide sugars: xylose and arabinose, although glucose or galactose may also be found in corn fiber [8,16,17]. The structure of AX in corn is that of a β-(1,4)-linked D-xylopyranose (Xyl*p*) backbone at C (O)-2 with 4-O-methyl-D-glucuronic acid and at C (O)-2 and/or C (O)-3 positions of α-L-arabinofuranose (Ara*f*) residues [5,7]. Most of the phenolic acid in CB is ferulic acid (FA), which is esterified to AX, but FA is localized at the C (O)-5 position of Ara*f*. Additionally, FA plays a significant role in the cross-linkages between polysaccharides containing AX and AX or AX and protein [18]. Moreover, although FA is proportionally the highest phenolic acid in AX, overall it is only a small portion of AX; nevertheless, FA tends to have significant effects on the molecular weight, solubility, and gelation properties of the AX in CB [19,20,21].

While CB and DDGS are used for animal feed, AX extracted from CB and DDGS can be utilized as the basis for materials in food packaging. People prefer food packaging to be transparent so that they can directly see what item can be bought or not [22]. A film created from AX is typically brittle, causing the film to split when handled; consequently, the film has low extensibility and flexibility [23,24]. Plasticizers, often sorbitol or glycerol, are typically used to reduce brittleness and to increase AX film’s flexibility, and both are recognized as safe compounds, humectants, and non-carcinogenic [23,24]. Sorbitol or glycerol used can significantly influence the characteristics of packaging materials. Whereas films containing glycerol have excellent mechanical properties such as extension and resistance, films made with sorbitol include a high hydrophobicity for food packaging materials that can improve the shelf-life of a packaged product [23,25].

The mechanical properties of AX films include tensile strength, tear resistance, and puncture resistance, which must be high to increase the strength of food packaging materials [26,27]. These mechanical characteristics of AX film are dependent on the AX source, plasticizer type, and the amount of plasticizer used [23,28,29]. The mechanical characterizations of AX films made from DCB, WCB, and DDGS for use in the food packaging industry have to be properly investigated to demonstrate the feasibility of film use for food packaging. Other properties of biomaterial films that must be considered are how they interact with water, such as the moisture content, water solubility, contact angle, water vapor transmission rate, and biodegradability. All the mechanical characteristics and water interaction properties of AX films determine their usability as a material in food packaging.

AX casting film can be modified with lipase to increase film hydrophobicity, with lipase suspended in vinyl acetate [30]. Thus, the use of lipase decreases the amount of acetate required for the esterification reaction [30,31]. Lipase has a high level of reaction specificity in hydrolyzing ester bonds, resulting in the esterification of AX [32,33,34]. The activity of lipase can demonstrate an esterified monosaccharide or oligosaccharide of AX, indicating a biomaterial film could be developed [30].

The objectives of this research were to extract AX and analyze AX characteristics from the three sources, including DCB, WCB, and DDGS, to include lipase–acetate application on the surface of AX casting films and to evaluate the mechanical properties of AX films. A biodegradable food packaging material was created using the combinations of AX solution, laccase, and sorbitol [28]; the surface of the casting AX film was modified by suspending it in acetate reagent with lipase in this research.

## 2. Materials and Methods

### 2.1. Materials and Size Reduction

Milled DCB was provided by Archer Daniels Midland (ADM) Company, Decatur, IL, USA. Corn bran produced through wet-milling, WCB was also provided by ADM, but this sample needed to be ground in the laboratory. DDGS were purchased from Elk Mound Seed Company, Elk Mound, WI. The DCB specimen had brown flour, while the WCB sample was milled to yellow flour using a UDY miller, cyclone sample mill from UDY corporation. The specimen of the DDGS was ground to sticky flour using the UDY miller.

### 2.2. Arabinoxylan Extraction

The three main steps of AX extraction from the milled DCB, WCB, and DDGS specimens included the following: defatting, acid–alkali extraction, and ethanol fractionation. Three samples were defatted by mixing DCB, WCB, or DDGS flour with hexane (1/10; *w*/*v*) [28]. The flour–hexane solution was twice stirred at 900 rpm, each stirring for 1 h at 25 °C using an LED Digital Stirrer. Arabinoxylan was extracted from the defatted DCB, WCB, and DDGS flour using 0.25 M hydrochloric acid (HCl) and 3.0 M sodium hydroxide (NaOH) before centrifuging to obtain solubilized arabinoxylan. A total of 50 grams (g) of defatted DCB, WCB, or DDGS was placed in 500 milliliters (mL) of 0.25 M HCl and stirred at 450 rpm on a hot plate set to 75 °C, holding the slurry temperature at 45 °C for 2 h. Then, 100 mL of 3.0 M NaOH was poured into the slurry while maintaining stirring and heating for another 2 h. The solution of the DCB, WCB, or DDGS specimen was neutralized to pH = 7 using concentrated HCl. The solution was then centrifuged at 4945 rpm for 10 minutes (min), and the supernatant was collected. Finally, 95% ethanol was added to the AX solution (2/1; *v*/*v*) and stirred for 1 h at 25 °C with 900 rpm in a Lab-Line Mistral Multi-Stirrer, and then the AX fraction was collected by centrifugation (6600 rpm, 10 min) before drying in an oven at 50 °C [14]. The weight of the AX pellet (from DCB, WCB, or DDGS) after each extraction was recorded, and the sample was stored in the freezer.

### 2.3. Arabinoxylan Composition and Characterization

The proximate compositions of the extracted AX were determined as follows: The moisture content of the three AX samples was determined by following the AACC International Method 44-15.02: Moisture Air-Oven Method [35]. The flour’s ash content of AX specimens was estimated following the AACC International Method 08-01.01: Ash-Basic Method [36]. The protein content of the AX flours was analyzed by estimating the nitrogen content with the AACC International Method 46-30.01: Crude Protein-Combustion Method [37]. The starch content of each AX specimen was analyzed by following the AACC International Method 76-13.01: a Megazyme Enzyme Assay Kit K-TSTA [38].

The sugar compositions of the AX yields were determined by High-Performance Anion-Exchange Chromatography–Pulsed Amperometric Detection (HPAEC-PAD). A 1 mL aliquot of 1 M HCl was separately added to 5–6 milligrams (mg) of each AX sample to ensure enough time for the removal of the acidic solvents by evaporation [39]. The samples were incubated in heating blocks at 100 °C for 1 h. The samples were cooled and neutralized using 1 mL of 1 M NaOH to facilitate the separation of Ara*f* and Xyl*p*. After that, the solutions were filtered through a 0.2 μm nylon syringe filter, and the sugar compounds of filtered samples were analyzed using HPAEC-PAD with a CarboPac PA20 column according to the method of a former study [39]. The sugar compositions of AX extracts from DCB, WCB, or DDGS were calculated according to the following formula:

(1) $$AX\left(\%\right)=0.88\times (\%Xylp+\%Araf)$$

The weight average molecular weights (Mw) and polydispersity index (PI) were determined by High-Performance Size-Exclusion Chromatography–Multi-Angle Light Scattering with Refractive Index (HPSEC-MALS-RI) [40]. The system was Shimadzu HPLC coupled to a Wyatt Opti lab RI detector and Wyatt MALS Dawn Heleos II. Separation was conducted using two columns (PL Aquagel-OH 40 and 60) connected in series at a flow rate of 0.5 mL/min. The mobile phase used was 0.05% sodium azide in double distilled water. The extracted AX of DCB, WCB, or DDGS was dissolved in 50 mM NaNO_3_ (2 mg/mL) for 16 h, and the solution was filtered through a 0.45 μm filter. A total of 100 μm of the sample was injected into the HPSEC-MALS-RI system. The dn/dc value was 0.146, and the Mw and PI were calculated using Astra 6.1.6 software. The light scattering model was Zimm with a fit degree of 1.

### 2.4. Modification of Arabinoxylan Films

**Figure 1:**
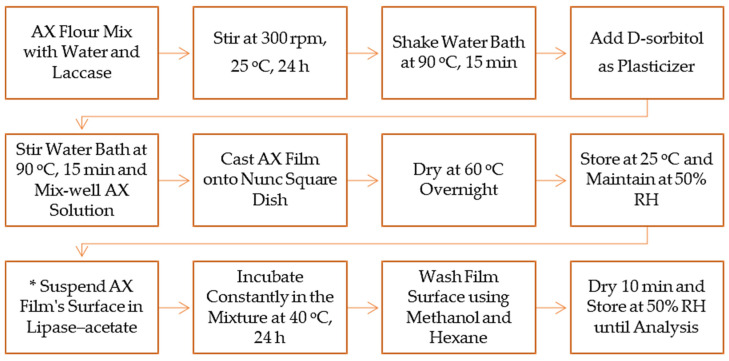
Flow chart of AX film casting. * Start modifying AX films.

AX film casting was completed in deionized water including D-sorbitol with a slight modification (Figure 1). The film solutions were created by making a 2.7% (*w*/*v*) AX extraction from DCB, WCB, and DDGS samples in deionized water with laccase from *Aspergillus* sp. Laccase (130 µL/2.7% AX film solution) added to produce cross-linked gel formations [19]. The AX solution was stirred for 24 h at 25 °C using a Cimarec i Poly 15 Stirrer. After one day, the solution was heated for 15 min at 90 °C in a water-shaking bath. D-Sorbitol (98% powder hygroscopic) was added as a plasticizer to the AX solution at 25% (*w*/*v*), 25% D-sorbitol/2.7% AX extraction [28]. After adding D-sorbitol, the solution was heated for 15 min at 90 °C in the water-shaking bath. Then, the AX film solutions made from DCB, WCB, and DDGS were cast onto a Nunc Square BioAssay Dish and dried at 60 °C overnight in an air oven (VWR^®^ Forced Air Ovens) (Figure 1). Once dried, all AX films were stored at Boveda 50–60% relative humidity (RH) and 25 °C in a dry keeper desiccator cabinet. The unmodified AX film from each sample had three replications. The modified AX film also included three replications. In order to modify the AX film, the surface of the AX film from the DCB, WCB, and DDGS samples was constantly incubated into a lipase–acetate mixture to increase film hydrophobicity [30]. The lipase was suspended in vinyl acetate, and the modified AX films were suspended in the reaction mixture at 40 °C for 24 h in a closed system [30]. On the following day, the surfaces of the modified AX films were washed several times using methanol and hexane and dried in the fume hood for 10 min (Figure 1). Finally, the modified AX films were maintained at 50–60% RH, 25 °C in the dry keeper desiccator cabinet before the determination of their mechanical properties and compared to their unmodified AX film counterparts.

### 2.5. Mechanical Properties of Biodegradable Arabinoxylan Films

#### 2.5.1. Morphology

The modified and unmodified AX films generated from DCB, WCB, and DDGS samples were dried in an oven at 105 °C for 1 h [41]. The dried AX film samples were cut into very small pieces (0.4 cm × 0.8 cm) and attached to the scanning electron microscope (SEM) stub with adhesive tape. A Nova nano SEM was utilized to obtain the images at 7.00 kilovolts (kV). Six morphology images were taken at magnification binoculars of 2022-2078x.

#### 2.5.2. Thickness

The thickness of each modified or unmodified AX film was measured by evaluating five points of the AX film’s surface using a Mitutoyo Dial Thickness Gauge, NO. 7304A. The surface of the AX film was inserted into the thickness gauge clipper, and the digital display was recorded in inches.

#### 2.5.3. Tensile Strength

**Figure 2:**
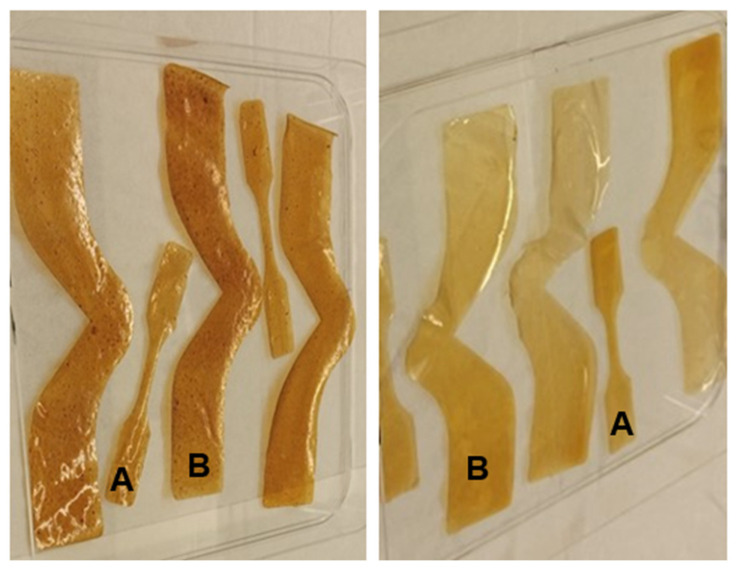
DCB AX films (**left**) and DDGS AX films (**right**) cutting by tear and tensile dies. (**A**) tensile shape. (**B**) tear shape.

The maximum tensile strength, percentage of tensile elongation, and modulus of elasticity were determined three times for the unmodified and modified AX films of the DCB, WCB, or DDGS specimen by a texture analyzer (Texture Technologies Group TA-XT2i). The peak tensile strength is the ratio between the maximum load and the minimum cross-sectional region of the film prior to testing. The percentage of tensile elongation indicates the ratio between the extension at break and the initial gage length multiplied by 100. The modulus of elasticity is the relationship between stress and strain in the load extension curve. This procedure was completed by following ASTM D882-18 [42]. The modified and unmodified AX film was cut using ASTM Specimen Die Pioneer Dietecs and VEVOR Leather Cutting Machine Manual Black (Figure 2A) [42]. The width of the breaking part (center) of the cutting film was 3.02 mm, and the break sensitivity was 750.2 millinewtons (mN) with tension test mode. The test temperature and RH of the cutting film were at 23–25 °C and 50–60%, respectively. The test speed and post-test speed were 8.30 and 10 mm/sec, respectively. The testing condition of tensile strength also included a strain rate of 51 mm and an initial grip separation of 25.4 mm. The AX film was held between initial grip separation settings: flat grip inserts and line grip inserts. The two grips completely cut the AX film into two pieces.

#### 2.5.4. Tear Resistance

The tear resistance of the modified and unmodified AX films made from DCB, WCB, and DDGS samples was determined three times using a texture analyzer (Texture Technologies Group TA-XT2i). This testing was performed by following ASTM D1004-21 [43]. Each AX film was cut using an ASTM D-1004 Tear Die and a VEVOR Leather Cutting Machine Manual Black (Figure 2B) [43]. The testing condition was at 23–25 °C and 50–60% RH of cutting film. The test mode was tension, with test speed and post-test speed 1 and 10 mm/sec, respectively. The trigger force was 49.0 mN, including a strain rate of 51 mm and an initial grip separation of 25.4 mm. The AX film was held between initial grip separation settings: flat grip inserts and line grip inserts. Once the test was completed, the two grips cut the film into two pieces. The tear resistance and tear extension outcomes were automatically calculated through the computer system.

#### 2.5.5. Puncture Resistance

Puncture resistance was conducted in three replications for each modified or unmodified AX film per sample using a texture analyzer (Texture Technologies Group TA-XT2i) with a 2 mm diameter stainless probe with a flat head. The puncture resistance of each modified or unmodified AX film was determined by a modified version of ASTM D7192-20 [44]. The test mode, test temperature, and RH of the 4 cm × 5 cm film piece were compression, at 23–25 °C, and 50–60%, respectively. The pre-test speed, test speed, and post-test speed were 2, 33, and 10 mm/s, respectively, with a 49.0 mN trigger force. To hold the AX films, a tortilla extensibility platform of the texture analyzer (Texture Technologies Group TA-XT2i) was utilized in conjunction via two CD disks with an outer diameter of 120 mm and an inner diameter of 15 mm [28]. The puncture resistance and puncture extensibility results were automatically calculated through Texture Exponent 32 software (Texture Technologies Corporation, Hamilton, MA, USA, 2016).

#### 2.5.6. Structural Analysis

Fourier-transform infrared (FTIR) spectroscopy was used to determine the interaction in structures between modified and unmodified AX films [45]. The testing condition was at 4 Reciprocal Centimeter (cm^−1^) resolution and 10 Aperture, with the range of the wavenumber between 4000 and 700 cm^−1^ (Thermo Scientific, Nicolet 8700, Wilmington, DE, USA).

### 2.6. Statistical Analysis

Statistical analysis was performed with SAS 9.4 for Windows, SAS Institute [46]. The source (DCB, WCB, and DDGS) was the first factor, and the modification of AX films was the second factor. For each dependent variable, we used two-way effects Model ANOVA to assess the main effects of the source and modification of AX films as well as their interaction. In all cases, the interaction was either not significant or if significant, it was of a diverging nature such that the main effects were still interpretable. Fisher’s least significant difference (LSD) was used for the mean separation of the levels of the main effects. A Cell Means model was used to assess differences among the interaction means, and the LSD was used for mean separation. All tests were assessed using the significant level of 0.05.

## 3. Results and Discussion

### 3.1. Proximate Compositions of Arabinoxylan Extractions

Extracted arabinoxylan (AX) was analyzed for its proximate composition, revealing significant variations among three samples: dry corn bran (DCB), wet corn bran (WCB), and distiller’s dried grains with solubles (DDGS), as shown in Table 1. The AX yields from DCB and DDGS were approximately 23.30% and 23.01%, respectively, while WCB accounted for a higher yield of 25.39% (Table 1). The differences in AX yields between DCB and DDGS were not statistically significant (*p* > 0.05); however, the yield from WCB was significantly higher (*p* < 0.05) compared to both DCB and DDGS. Arabinoxylan is primarily extracted from corn bran (CB), where its concentration typically ranges from 25 to 35% of the total bran content [47,48]. The elevated yield in WCB can be attributed to differences in processing methods. Corn dry-milling separates the kernel’s main physical components—germ, bran, and endosperm [1]—whereas wet-milling divides the grain’s primary chemical compositions into starch, protein, fiber, and oil [4]. The ethanol production process, which includes additional steps like fermenting, distilling, and centrifuging, complicates the extraction of AX from DDGS [14].

Moisture content is commonly used as a primary metric for evaluating other proximate compositions in food science research [49,50]. In our study, the AX extracted from DCB had the highest moisture percentage at 14.14%, followed by the AX from DDGS at 13.85%, and the AX from WCB at 13.15% (Table 1). This indicates that the moisture contents of the AX yields from these three sources are significantly different (*p* < 0.05), suggesting that variations in moisture content among the AX yields from DCB, WCB, and DDGS samples could be attributed to differences in material sources and storage conditions.

The concentration of AX is influenced by the mill stream, and a notable correlation has been observed between ash and AX contents, indicating that samples with a lower ash content tend to have lower AX content [51]. In this context, the AX from DDGS exhibited the highest ash content at 18.36%, markedly higher than that found in DCB AX and WCB AX, which had ash contents of 5.36% and 5.86%, respectively (Table 1). Therefore, the ash contents of all AX extracts were significantly different from one another (*p* < 0.05), supporting the premise that a higher ash content correlates with higher AX content.

Protein plays a critical role in forming the structure of cell walls in the corn kernel [1,52]. The acid–alkali extraction process for AX, which involves disrupting covalent and hydrogen bonds and breaking down the corn bran matrix, results in extracts low in protein and starch [53]. The protein contents in DCB AX and DDGS AX were 6.67% and 6.51%, respectively, both significantly lower (*p* < 0.05) than the 9.33% protein content found in the WCB AX extract (Table 1). Similarly, the starch content in WCB AX and DDGS AX was approximately 2.5% for each, significantly lower (*p* < 0.05) than the 8.83% starch content in DCB AX (Table 1). The relatively low starch contents in WCB and DDGS are reflected in their AX extracts, likely a consequence of the chemical extraction method’s hydrolyzing effect on starch, leading to a decrease in starch content in WCB AX and DDGS AX due to the release of more non-polysaccharides through acid–alkali extraction [16,53].

### 3.2. Arabinoxylan Characterizations

The concentration of AX and its arabinose to xylose ratio (A/X) differ based on the source of the byproduct or coproduct and the extraction procedure employed. For the AX extracted from DCB, WCB, and DDGS, the polymer percentages were 76.60%, 77.64%, and 66.68%, respectively, as outlined in Table 1. Additionally, the A/X ratios for these extracts were distinct, measuring at 1.02 for DCB AX, 1.20 for WCB AX, and 1.32 for DDGS AX, also noted in Table 1. These differences in AX polymer composition and A/X ratios among the three types of extracted AX were statistically significant (*p* < 0.05), highlighting the variability in their chemical structures.

Regarding the Mw, the AX extracts varied significantly (*p* < 0.05), with DCB AX showing the highest molecular weight at 4,063,667, followed by WCB AX at 1,876,333, and DDGS AX at 879,133, as indicated in Table 1. This trend suggests that a higher AX concentration correlates with a greater Mw, a concept supported by previous findings [32]. The two Mws observed in this study are consistent with this hypothesis, demonstrating a clear relationship between the AX content and Mw of the extracts from WCB and DDGS. Furthermore, a low A/X ratio leads to low AX solubility, making it an increase in the Mw of AX [48]. Because of a lower A/X ratio and reduction in AX solubility, the Mw of AX extracted from DCB was much larger compared to the two molecular weights.

Similarly, the polydispersity index (PI) for all AX extracts was significantly different (*p* < 0.05) across the different AX yields, with DCB AX having the highest PI at 3.05, followed by WCB AX at 2.37, and DDGS AX at 1.80, as shown in Table 1. Since all PIs were above 1, it indicates that the molecular weights of the AX extracts are greater than their number average molecular weights [54]. This variation in the PI underscores the heterogeneity present in the AX extracts derived from DCB, WCB, and DDGS, reflecting the complexity and diversity of their structural properties.

### 3.3. Arabinoxylan Film Morphology

**Figure 3:**
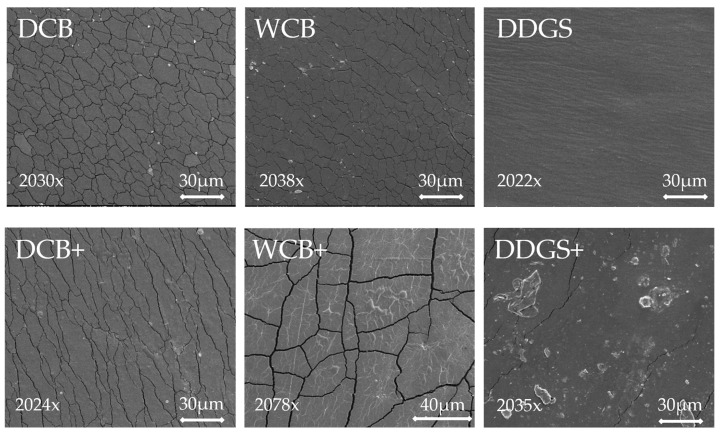
Scanning electron microscopy (SEM) of unmodified and modified arabinoxylan (AX) films. Dry corn bran: DCB; wet corn bran: WCB; distiller’s dried grains with solubles: DDGS. +: AX film that suspended lipase–acetate.

The surface morphology of both modified and unmodified AX films derived from DCB, WCB, and DDGS was meticulously examined using scanning electron microscopy (SEM). Through SEM, the evaporated surface images of the AX films exhibited distinctive topographical features and morphological structures, which manifested as variations in brightness or color intensity in the images (Figure 3). Specifically, bright spots within these images indicated the presence of nickel, a common practice in SEM analysis for enhancing image contrast. These bright spots were observed in both unmodified and modified AX films from DCB and WCB samples. Notably, the modified AX film from DDGS samples featured the largest bright spots among the examined samples (Figure 3).

The presence of cracks on the surface of these AX films is attributed to the drying process in an oven, a necessary step in preparing the solid surface of the sample before SEM analysis [41]. This drying process can induce stress on the film’s surface, leading to crack formation. Interestingly, the modified AX films from DCB and WCB exhibited fewer surface cracks compared to their unmodified counterparts (Figure 3), suggesting that modification processes may influence the film’s structural integrity. It is plausible that fewer cracks are indicative of a smoother or more impermeable film surface, while a higher prevalence of cracks may correlate with a rougher or more porous film structure [55]. A relationship between film morphology and water solubility was found in this study [55]. Cracks might be an inlet for water in the inside of the compound matrix, resulting in a soluble film, which is reinforcing for biodegradable properties.

Furthermore, the unmodified AX films from DDGS samples displayed no visible cracks, yet they possessed a rough surface texture, which could imply greater permeability. Conversely, the modified AX films from DDGS were characterized by small cracks and the inclusion of large spots, a feature that might suggest a tendency towards impermeability. This variance in surface morphology between modified and unmodified AX films across different sample sources provides insight into how modification processes can affect the physical properties of AX films, potentially impacting their application in various industries.

### 3.4. Arabinoxylan Film Thickness

The thickness of AX films varied significantly among samples derived from DCB, WCB, and DDGS, a variance attributable to the differing sources of the samples and the inherent heterogeneity of AX [55]. Specifically, the AX film produced from DCB exhibited the greatest thickness, measuring 0.0041 inches, which was considerably thicker than the AX films originating from WCB and DDGS, with thicknesses of 0.0036 inches and 0.0037 inches, respectively (Table 2). These findings align with the thickness ranges reported in prior studies, reinforcing the impact of the source material and AX composition on film thickness [55,56].

**Figure 4:**
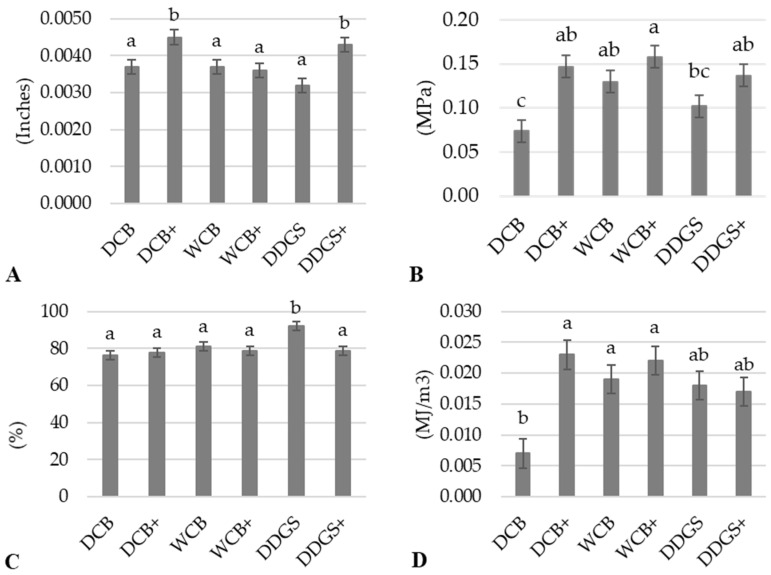
Interactions of thickness and tensile properties of unmodified and modified AX films. (**A**) Thickness. (**B**) Tensile strength. (**C**) Tensile elongation. (**D**) Modulus of elasticity. Means with the same letter in the same sub-figure are not significantly different (*p* > 0.05). The data are the means of three independent replicate experiments (n = 3). Thickness (inches); tensile strength (megapascals = MPa); tensile elongation (%); modulus of elasticity (megajoule per cubic meter = MJ/m^3^). Dry corn bran: DCB; wet corn bran: WCB; distiller’s dried grains with solubles: DDGS. +: AX film that suspended lipase–acetate.

Moreover, the thickness of the modified AX film samples, for all three sources, was uniformly measured at 0.0041 inches, marking a significant increase (*p* < 0.05) compared to the 0.0035 inches thickness of the unmodified AX films (Table 2). Particularly, this increase in thickness was notably significant (*p* < 0.05) for the modified AX films derived from DCB and DDGS when juxtaposed with their unmodified counterparts (Figure 4A). Such an increase in thickness for the modified films from DCB and DDGS can be attributed to the addition of a lipase–acetate mixture, suggesting that this modification process enhances the structural integrity of the AX films.

Conversely, the modified AX film produced from WCB demonstrated a thickness comparable to that of its unmodified version, with no statistically significant difference between the two (*p* > 0.05) (Figure 4A). This observation might suggest that AX films from WCB inherently possess a minimal thickness that remains relatively unaffected by the modification process, possibly due to the unique composition of WCB-derived AX. This characteristic indicates that while modifications can influence the thickness and potentially the physical properties of AX films from DCB and DDGS, the effect on WCB-derived films appears to be more subdued, underscoring the complex interplay between the source material, modification processes, and the resulting physical characteristics of AX films.

### 3.5. Tensile Strength

The tensile properties of film sheeting, crucial for the evaluation of food packaging materials, are significantly influenced by the film’s preparation method, testing speed, and thickness. These properties, including tensile strength, tensile elongation, and the modulus of elasticity, are essential parameters for assessing the suitability of packaging materials, which require a high tensile strength and modulus of elasticity to enhance their tensile properties [55]. A balanced combination of tensile strength and tensile elongation is vital for developing the requisite durability in food packaging materials [57].

In our study, the AX film derived from WCB exhibited the highest tensile strength at 0.144 MPa, followed by the AX film from DDGS at 0.120 MPa, and the AX film from DCB at 0.111 MPa, as detailed in Table 2. Similarly, the WCB AX film demonstrated the greatest modulus of elasticity at 0.020 MJ/m^3^, with the DDGS AX film and the DCB AX film following at 0.017 MJ/m^3^ and 0.015 MJ/m^3^, respectively (Table 2). Although there were no significant differences (*p* > 0.05) observed between the sources of the AX film regarding their elastic modulus and tensile strength, the results for the WCB AX film suggest that it is more ductile, bendable, and resistant to deformation when stretched compared to the AX films from DCB and DDGS. This finding aligns with desirable characteristics for ductile and bendable packaging materials [55,56].

The tensile elongation was notably different, with the DCB AX film showing the lowest percentage at 77.01%, which was significantly lower (*p* < 0.05) than the 85.47% observed for the DDGS AX film (Table 2). A high percentage of tensile elongation is indicative of a material’s ability to deform elastically when stretched, an essential feature for certain packaging applications [55].

Furthermore, an interesting observation was made regarding the tensile strength of modified AX films. When the surfaces of these films were treated with a lipase–acetate solution, a significant increase in tensile strength was noted, rising to 0.148 MPa for the modified films compared to 0.102 MPa for the unmodified samples (Table 2). Although the modulus of elasticity for the modified AX films increased to 0.021 MJ/m^3^ from 0.015 MJ/m^3^ for the unmodified films, this change was not statistically significant (*p* > 0.05) (Table 2). The tensile elongation decreased slightly in the modified films to 78.31% from 83.28% in the unmodified films (Table 2), yet this difference was also not significant (*p* > 0.05).

These results indicate that modifying the surface of AX films enhances their tensile properties, rendering the AX film sheeting more ductile and resistant to deformation. This improvement underscores the potential for modified AX films in food packaging applications, where robust mechanical properties are a prerequisite for performance and durability.

The statistical analysis revealed a significant increase (*p* < 0.05) in the tensile strength of the modified arabinoxylan (AX) film derived from dry corn bran (DCB) when compared to its unmodified counterpart (Figure 4B). This enhancement in tensile strength was not mirrored in the modified AX films from wet corn bran (WCB) and distiller’s dried grains with solubles (DDGS), where improvements were observed but did not reach statistical significance (*p* > 0.05) compared to their respective unmodified films (Figure 4B). This suggests that the modification process, involving suspension in a lipase–acetate mixture, has a distinct impact on the tensile strength of AX films, contributing to the development of more flexible films. This observation aligns with findings from other contemporary research [55,56], highlighting the potential benefits of enzymatic–chemical modification in enhancing film properties.

Furthermore, the modified DCB AX film displayed a significant increase (*p* < 0.05) in the elastic modulus compared to the unmodified DCB AX film (Figure 4D), corroborating the observed improvement in tensile strength. While the modified WCB AX film also showed an increase in its elastic modulus, this change was not statistically significant (*p* > 0.05) when compared to its unmodified version (Figure 4D). The enhancement in both the tensile strength and elastic modulus in modified AX films suggests their suitability as flexible and bendable materials for food packaging applications.

Interestingly, the tensile elongation for the modified DCB AX and WCB AX films remained similar to that of their unmodified samples, with no statistically significant differences observed between them (*p* > 0.05) (Figure 4C). This indicates that the modification process does not adversely affect the film’s ability to elongate under tension. In contrast, the modified DDGS AX film exhibited a significant reduction (*p* < 0.05) in the percentage of tensile elongation compared to the unmodified DDGS AX film (Figure 4C). Such a decrease in tensile elongation could result in films that are less deformable, potentially limiting their application in certain types of food packaging where flexibility is desired, as also documented in the existing literature [55]. This nuanced understanding of how modifications influence the tensile properties of AX films underscores the complexity of optimizing film characteristics for specific packaging needs.

### 3.6. Tear Resistance

In the evaluation of tear resistance, crucial factors such as film thickness, film geometry, and the speed of testing are meticulously controlled. This method aims to identify the force required to initiate a tear across a specified film geometry, with the peak stress encountered being quantified as the tear resistance [24]. Consequently, the maximum tear resistance and the maximum tear extension are computed for the film samples tested at a predetermined speed. Ideal food packaging materials should exhibit high tear resistance coupled with low tear extension to optimize their tear characteristics [27]. The load necessary before tear propagation commences is also considered an indicator of the film sheeting’s resistance to rupturing. Essentially, a greater load enhances tear resistance and diminishes tear extension.

Among the tested samples, the AX film derived from DCB showcased the highest tear resistance, recorded at 1.05 N, followed by the AX film from WCB at 0.85 N, and the AX film from DDGS at 0.40 N (Table 3). The disparity in tear resistance between the DCB AX and WCB AX films was not statistically significant (*p* > 0.05); however, both showed a significant difference (*p* < 0.05) when compared to the tear resistance of the DDGS AX film (Table 3). As a consequence, the DCB AX film demonstrated superior flexibility in packaging film sheeting, in contrast to the DDGS AX film, which exhibited reduced flexibility. Bio-based films that are characterized by a higher tear resistance and lower extensibility are deemed suitable for flexible and pliable food packaging applications [26].

Moreover, the DDGS AX films exhibited the greatest tear extensibility at 57.69 mm, with the DCB AX films and WCB AX films following closely at 56.31 mm and 55.90 mm, respectively (Table 3). No significant difference (*p* > 0.05) was observed in tear extension among the AX film sources. A reduction in the extensible behavior of the WCB AX films was associated with increased material flexibility.

The tear resistance of both modified and unmodified AX films was observed to increase following the immersion of AX film surfaces in a lipase–acetate solution. Specifically, the tear resistance for unmodified AX films was significantly enhanced from 0.39 N to 1.14 N for the modified AX films (*p* < 0.05) (Table 3). Similarly, the tear extension for both film types showed a decrease after treatment with the lipase–acetate mixture. The tear extension of unmodified AX films, initially at 57.82 mm, was significantly reduced to 55.45 mm for the modified AX films (*p* < 0.05) (Table 3). This trend suggests that bio-based films with a high tear resistance and low extensibility are optimal for creating flexible and pliable food packaging materials [26,27]. The modifications applied to the AX film surfaces in this study successfully enhanced their tear strength, thereby increasing the flexibility and pliability of the AX film materials, indicating a promising advancement for food packaging technology.

Maintaining a uniform film thickness within the same sample is crucial for the validity of tear resistance comparisons, as variations in thickness can significantly influence tear resistance measurements [26]. The investigation into the thickness of unmodified and modified WCB AX films revealed no significant difference (*p* > 0.05), ensuring a reliable basis for comparing their tear resistance (as indicated in Figure 4A). This uniformity in thickness set the stage for observing significant differences in the tear resistance and extensibility behaviors between the modified and unmodified WCB AX films.

**Figure 5:**
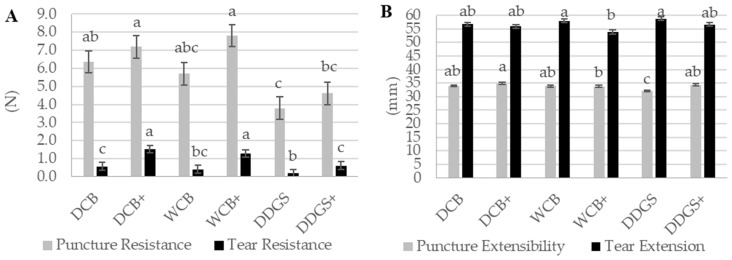
Interactions of tear and puncture resistances of unmodified and modified AX films. (**A**) Tear and puncture resistances. (**B**) Tear and puncture extensibilities. Means with the same letter in the same sub-figure are not significantly different (*p* > 0.05). The data are the means of three independent replicate experiments (n = 3). Tear and puncture resistances (newton = N); tear and puncture extensibilities (millimeter = mm). Dry corn bran: DCB; wet corn bran: WCB; distiller’s dried grains with solubles: DDGS. +: AX film that suspended lipase–acetate.

Notably, the tear resistance of the modified WCB AX film exhibited a significant increase (*p* < 0.05) compared to its unmodified counterpart (Figure 5A), demonstrating the efficacy of the modification process in enhancing this critical property. Additionally, the tear extension of the modified WCB AX films showed a significant decrease (*p* < 0.05) compared to the unmodified films (Figure 5B). These findings collectively highlight the modification process’s success in optimizing the tear strength of WCB AX films, resulting in materials with improved flexibility suited for packaging applications [26].

Similarly, the modified DCB AX and DDGS AX films also showed significant improvements in tear resistance (*p* < 0.05) when compared to their unmodified versions (Figure 5A). However, unlike the WCB AX films, the tear extension for the modified DCB AX and DDGS AX films did not significantly change (*p* > 0.05) (Figure 5B), indicating that while their resistance to tearing increased, their ability to extend before breaking remained consistent with the unmodified films.

This enhancement in tear resistance across all modified AX films, attributed to the treatment with a lipase–acetate solution, underscores the positive impact of surface modification on the mechanical properties of AX films. By achieving a balance between tear resistance and extension, the modified films emerge as more flexible and suitable candidates for food packaging materials, aligning with the objectives of creating durable, reliable packaging solutions.

### 3.7. Puncture Resistance

Puncture resistance is a crucial factor in the selection of materials for food packaging, influenced by the type of polymer composition, film fabrication procedure, and film uniformity. Defined as a material’s ability to withstand a concentrated force over a small area, puncture resistance is vital due to the stresses food packaging endures from industry to consumer transport. Notably, both the extraction level and purity of AX can enhance the puncture resistance of AX films [28]. In contrast to tear resistance evaluations, comparing puncture resistance does not necessitate uniform film thicknesses for films derived from the same AX polymer.

In this study, the puncture resistance values for DCB AX and WCB AX films were similar and not statistically different (*p* > 0.05), both estimated at approximately 6.75 N (Table 3). However, the puncture resistance of the DDGS AX film was significantly lower, recorded at 4.21 N (*p* < 0.05). This difference suggests that the higher AX extraction from DCB and WCB contributes to their films’ increased puncture resistance, whereas the lower AX yield from DDGS results in diminished resistance [28]. Further, the purity of AX from corn bran has been linked to higher puncture resistance in AX films, with reduced AX extraction from DDGS correlating with decreased resistance [28].

The puncture extensibility observed in the DCB AX film was 34.43 mm, significantly higher (*p* < 0.05) than the 33.17 mm measured for the DDGS AX film (Table 3). The WCB AX film’s puncture extensibility was 33.78 mm, showing no significant difference (*p* > 0.05) from the other samples, suggesting an increase in puncture resistance may correspond with increased extensibility, a trend supported by previous studies [28,58].

Modifications through suspending AX film surfaces in a lipase–acetate solution led to an increase in both puncture resistance and extensibility. The puncture resistance for modified AX films was slightly higher at 6.54 N compared to 5.29 N for unmodified films, though this increase was not statistically significant (*p* > 0.05). However, the puncture extensibility of modified films significantly improved to 34.29 mm from 33.29 mm for unmodified films (*p* < 0.05) (Table 3), indicating that film composition and formation processes can affect these properties [28,58]. Consequently, surface modification enhances puncture resistance, yielding more ductile and flexible films suitable for food packaging.

No significant interaction was noted for the puncture resistance between modified and unmodified AX films from the same source, although all modified films showed increased resistance. Interestingly, a relationship was observed between surface film cracks, detected through scanning electron microscopy (SEM), and puncture resistance. Unmodified DCB AX and WCB AX films exhibited more surface cracks and consequently lower puncture resistance, failing to maintain ductile, elastomeric properties. Conversely, modified DCB AX and WCB AX films, with fewer surface cracks, demonstrated improved puncture resistance. This correlation underscores that films with fewer surface cracks and higher puncture resistance are more adaptable and suitable for use in food packaging. Significantly, only the puncture extensibility of modified versus unmodified DDGS AX films showed a significant difference (*p* < 0.05), highlighting that lipase–acetate preparation contributes to both higher puncture resistance and extensibility.

### 3.8. Structural Analysis

**Figure 6:**
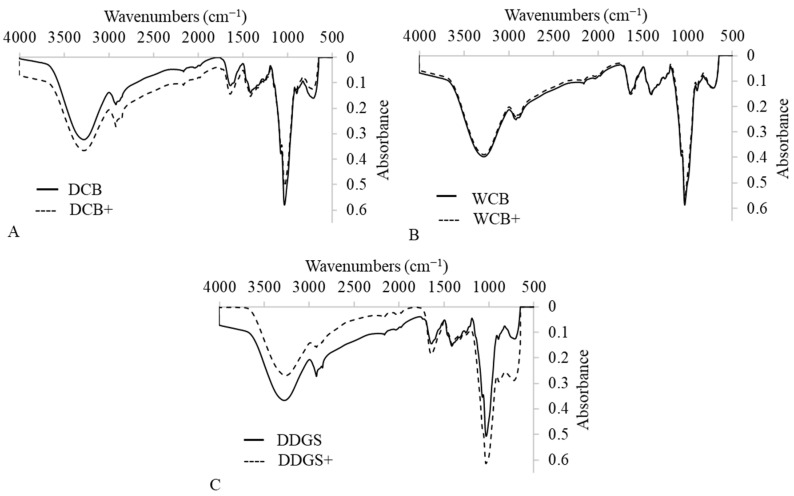
FTIR spectroscopy profiles for unmodified and modified arabinoxylan (AX) films made with dry corn bran: DCB (**A**), wet corn bran: WCB (**B**), and dried distiller’s grains with solubles: DDGS (**C**).

A Fourier-transform infrared spectroscopy (FTIR) analysis was conducted to identify the chemical compounds and functional groups within AX films, which are pivotal in determining the suitability of these materials for food packaging applications. The FTIR spectra for both modified and unmodified AX films derived from DCB, WCB, and DDGS encompassed a broad range from 600 cm^−1^ to 3700 cm^−1^, showcasing the diverse chemical composition of these films (Figure 6A–C). A signal ranging from 3265 cm^−1^ to 3277 cm^−1^ was attributed to O−H stretching, indicative of hydroxyl groups within the AX films, a common feature across all samples (Figure 6A–C). This presence of hydroxyl groups is crucial as it points to the films’ hydrophilic nature.

Furthermore, the spectra revealed C=O stretching vibrations between 1637 cm^−1^ and 1642 cm^−1^, identifying the existence of carbonyl groups (Figure 6A–C). The detection of these groups is significant for understanding the chemical functionality of AX films. Additionally, characteristic bands appearing from 2917 cm^−1^ to 2935 cm^−1^ demonstrated C−H stretching, while bands from 1407 cm^−1^ to 1413 cm^−1^ were indicative of C−H bending in the AX films, aligning with findings from previous research by Zhang et al., 2011 [59].

The analysis not only confirmed the presence of hydroxyl (OH) and carbonyl (CO) groups but also identified ester compounds within the AX films. The occurrence of OH, CO, and ester compounds in these plant-based films suggests excellent biodegradability properties [60,61], an essential characteristic for sustainable food packaging solutions. Notably, the absorption of OH, CO, and ester compounds in the modified DCB AX films was higher than in their unmodified counterparts (Figure 6A), indicating an alteration in chemical structure post-modification. Conversely, the absorption levels of these functional groups in the modified WCB AX films showed no significant difference compared to the unmodified WCB AX films (Figure 6B). The modified DDGS AX films, however, exhibited a decrease in OH absorption and an increase in CO and ester compounds compared to the unmodified DDGS AX films (Figure 6C), reflecting changes in chemical composition due to the modification process.

Overall, these findings underscore the impact of suspension in a lipase–acetate solution on the chemical composition of DCB AX and DDGS AX films, enhancing their biodegradability properties. This adjustment in functional group concentrations, particularly in modified films, provides insights into the potential for these materials to serve as environmentally friendly options in food packaging, aligning with the growing demand for sustainable packaging solutions.

## 4. Conclusions

The modification of AX films (derived from DCB and DDGS) through immersion in a lipase–acetate mixture resulted in an increase in film thickness, highlighting the efficacy of this treatment in enhancing the physical structure of the films. Contrarily, the AX film from wet corn bran (WCB) maintained a consistent thickness post-modification, indicating a varied response to the treatment based on the source material. Moreover, the tensile properties of the modified AX films from DCB and DDGS experienced a significant enhancement compared to their unmodified counterparts. The modified AX films derived from WCB also showed improved tensile properties, albeit insignificant improvement. These enhancements emphasize the impact of the lipase–acetate suspension in augmenting the films’ mechanical strength, making them more ductile, bendable, and resistant to deformation when stretched.

The surface modification process applied to AX films from all three sources (DCB, WCB, and DDGS) notably increased their tear resistance, affirming a boost in material flexibility and pliability. While puncture resistance saw an uplift in the modified films as well, the change was not statistically significant, suggesting a nuanced influence of the modification on this particular property. Collectively, these modifications have rendered the AX films with heightened mechanical properties—namely tensile strength, tear tolerance, and puncture resistance—thereby enhancing their suitability as robust food packaging materials. Among these, the modified AX film derived from DCB exhibited superior physical and mechanical characteristics compared to films made from WCB and DDGS.

Regarding further analysis, the color variation observed in the modified AX films suggests an avenue for additional research, particularly given the importance of aesthetic appeal in food packaging for marketing purposes. The observed color differences—darker, brown shades in DCB AX films versus lighter, more yellow hues in WCB AX films—point to the potential for tailoring the visual attributes of packaging materials to enhance consumer appeal. The color of AX films can be precisely evaluated using instruments such as the MacBeth Color Eye, employing color measurement standards like CIELab and Hunter Lab [55]. However, there is a noticeable gap in the literature concerning the impact of lipase–acetate incubation on the yellow and white properties of biodegradable films derived from corn bran AX.

Future research directions should encompass a broad spectrum of studies to further enhance the utility and acceptance of AX films in food packaging. These should include investigations into the long-term color stability of modified AX films under various environmental conditions and the exploration of methods for intentional color modification to align with branding strategies. Additionally, comprehensive studies on the biodegradability rates of these films in different settings, consumer perception analysis focusing on color and mechanical properties, and the enhancement of barrier properties such as moisture, oxygen, and lipid permeability are crucial. Finally, assessing the scalability of modified AX film production for commercial use, including cost analysis, manufacturing process optimization, and regulatory compliance, will be vital for their practical application in the food packaging industry. Collectively, these research avenues aim to not only improve the functional and aesthetic properties of AX films but also to ensure their sustainability and market acceptance.

## Figures and Tables

**Table 1:** Proximate composition and characteristics of arabinoxylan extractions.

Extracted AX	Yield	Moisture	Ash	Protein	Starch	AX	A/X	Mw	PI
DCB	23.30 a	14.14 a	5.36 a	6.67 a	8.83 a	76.60 a	1.02 a	4,063,667 a	3.05 a
WCB	25.39 b	13.15 b	5.86 b	9.33 b	2.53 b	77.64 b	1.20 b	1,876,333 b	2.37 b
DDGS	23.01 a	13.85 c	18.36 c	6.51 a	2.73 b	66.68 c	1.32 c	879,133 c	1.80 c

a–c Means with the same letter in the same column for the same sample type are not significantly different (*p* > 0.05). The data are the means of three independent replicate experiments (n = 3). Yield, moisture, ash, protein, starch, and AX (Dry Weight Basis = DWB%); A/X: arabinose to xylose ratio; Mw: weight average molecular weight; PI: polydispersity index; AX and A/X are the sugar measurement of the AX yields by HPAEC-PAD. Mw and PI are the molecular weight characteristics of AX yields using HPSEC-MALS-RI. Dry corn bran: DCB; wet corn bran: WCB; distiller’s dried grains with solubles: DDGS.

**Table 2:** Evaluation of thickness and tensile properties of unmodified and modified AX films.

Factors	AX Films	Thickness	Tensile Strength	Tensile Elongation	Modulus of Elasticity
Sources	DCB	0.0041 a	0.111 a	77.01 a	0.015 a
	WCB	0.0036 b	0.144 a	79.91 ab	0.020 a
	DDGS	0.0037 ab	0.120 a	85.47 b	0.017 a
Modification	Unmodified	0.0035 a	0.102 a	83.28 a	0.015 a
	Modified	0.0041 b	0.148 b	78.31 a	0.021 a

a,b Means with the same letter in the same column for the same factor are not significantly different (*p* > 0.05). The data are the means of three independent replicate experiments (n = 3). Thickness (inches); tensile strength (megapascals = MPa); tensile elongation (%); modulus of elasticity (megajoule per cubic meter = MJ/m^3^). Dry corn bran: DCB; wet corn bran: WCB; distiller’s dried grains with solubles: DDGS.

**Table 3:** Determination of tear and puncture properties of unmodified and modified AX films.

Factors	AX Films	Tear Resistance	Tear Extension	Puncture Resistance	Puncture Extensibility
Sources	DCB	1.05 a	56.31 a	6.78 a	34.43 a
	WCB	0.85 a	55.90 a	6.75 a	33.78 ab
	DDGS	0.40 b	57.69 a	4.21 b	33.17 b
Modification	Unmodified	0.39 a	57.82 a	5.29 a	33.29 a
	Modified	1.14 b	55.45 b	6.54 a	34.29 b

a,b Means with the same letter in the same column for the same factor are not significantly different (*p* > 0.05). The data are the means of three independent replicate experiments (n = 3). Tear and puncture resistances (newton = N); tear and puncture extensibilities (millimeter = mm). Dry corn bran: DCB; wet corn bran: WCB; distiller’s dried grains with solubles: DDGS.

## Data Availability

The original contributions presented in the study are included in the article, further inquiries can be directed to the corresponding author.
